# Relation between Red Cell Distribution Width and Fibroblast Growth Factor 23 Cleaving in Patients with Chronic Kidney Disease and Heart Failure

**DOI:** 10.1371/journal.pone.0128994

**Published:** 2015-06-16

**Authors:** Fenna van Breda, Mireille E. Emans, Karien van der Putten, Branko Braam, Frans J. van Ittersum, Rob J. Kraaijenhagen, Martin H. de Borst, Marc Vervloet, Carlo A. J. M. Gaillard

**Affiliations:** 1 Department of Nephrology and ICaR-VU, VUMC, Amsterdam, the Netherlands; 2 Department of Cardiology, Ikazia Hospital, Rotterdam, the Netherlands; 3 Department of Internal Medicine, TerGooi Hospital, Hilversum, the Netherlands; 4 Department of Medicine, division of Nephrology and Immunology, University of Alberta, Edmonton, Canada; 5 Department of Clinical Chemistry, Meander Medical Center, Amersfoort, the Netherlands; 6 Department of Nephrology, UMCG, Groningen, the Netherlands; Institut national de la santé et de la recherche médicale (INSERM), FRANCE

## Abstract

**Objective:**

In chronic kidney disease (CKD), both anemia and deregulated phosphate metabolism are common and predictive of adverse outcome. Previous studies suggest that iron status influences phosphate metabolism by modulating proteolytic cleavage of FGF23 into C-terminal fragments. Red cell distribution width (RDW) was recently identified as a strong prognostic determinant for cardiovascular morbidity and mortality, independently of iron status. We assessed whether RDW is associated with FGF23 cleaving in CKD patients with heart failure.

**Materials and Methods:**

The associations between RDW and either intact FGF23 (iFGF23), C-terminal FGF23 (cFGF23, reflecting iFGF23 and C-terminal fragments together) and the iFGF23/cFGF23 ratio were analyzed in 52 patients with CKD (eGFR 34,9 ± 13.9 ml/min/1.73m^2^) and chronic heart failure (CHF). Associations between RDW and FGF23 forms were studied by linear regression analysis adjusted for parameters of renal function, iron metabolism, phosphate metabolism and inflammation.

**Results:**

Median cFGF23 levels were 197.5 [110–408.5] RU/ml, median iFGF23 levels were 107.3 [65.1–162.2] pg/ml and median FGF23 ratio was 0.80 [0.37–0.86]. Mean RDW was 14.1 ± 1.2%. cFGF23 and RDW were associated (β = 1.63x10^-3^, P <0.001), whereas iFGF23 and RDW were not (β = -1.38x10^-3,^ P = 0.336). The iFGF23/cFGF23 ratio was inversely associated with RDW. The difference between cFGF23 and iFGF23 (cFGF23- iFGF23) was positively associated with RDW (β = 1.74x10^-3^, P< 0.001). The association between cFGF23 and RDW persisted upon multivariable linear regression analysis, adjusted for parameters of renal function, phosphate metabolism, iron metabolism and inflammation (β = 0.97x10^-3^, P = 0.047).

**Conclusion:**

RDW is associated with cFGF23 but not with iFGF23 levels in patients with CKD and CHF. This suggests a connection between RDW and FGF23 catabolism, independent of iron status and inflammation. Future studies are needed to unravel underlying mechanisms and whether these pertain to the link between RDW and outcome.

## Introduction

The simultaneous occurrence of chronic heart failure (CHF) and chronic kidney disease (CKD), known as the cardiorenal syndrome (CRS), is accompanied by high morbidity and mortality [1,2]. Traditional risk factors only partly explain this high risk [3], suggesting that additional pathophysiological mechanisms are involved. Several novel risk factors have been implicated in the elevated cardiovascular risk in CKD. Prominent non-traditional risk factors include red cell related measures such as anemia, iron status and red cell distribution width (RDW) [4], and markers of mineral metabolism, especially fibroblast growth factor 23 (FGF23) [5]. Interestingly, recent studies suggest a mechanistic link between these two systems [6–8].

FGF23 is a bone derived phosphaturic hormone that plays an important role in systemic phosphate homeostasis and vitamin D metabolism. Several observational studies consistently demonstrate independent associations between FGF23 and accelerated CKD progression [9], left ventricular hypertrophy in dialysis and predialysis patients [10], and increased mortality risk in CKD and hemodialysis patients and kidney transplant recipients [10–14]. Recently, it was shown that iron status influences FGF23 catabolism in mice with autosomal dominant hypophosphatemic rickets [6]. Similarly, in female patients with iron deficient anemia markedly elevated C-terminal FGF23 (cFGF23) levels but not intact FGF23 (iFGF23) levels were found [7]. Importantly, intravenous iron administration markedly reduced cFGF23 levels, providing another clue that iron status influences FGF23 cleaving. The current hypothesis is that, in healthy individuals, iron deficiency stimulates FGF23 production whereby osteocytes couple increased production of FGF23 with increased cleavage to cFGF23 to maintain normal circulating levels of iFGF23, which is the biologically intact hormone [15]. However, it is unknown whether this finding holds for CKD, a disease characterized by disturbed iron metabolism, high FGF23 levels and increased risk for cardiovascular complications.

Red cell distribution width (RDW) is a measure of the variation of red blood cell volume, defined as the standard deviation of erythrocyte size divided by the mean corpuscular volume. RDW is a robust marker of adverse clinical outcomes in patients with chronic and acute heart failure [16–19], coronary artery disease [20], acute kidney injury (AKI) [21] and even in the community [22–24]. The pathophysiological mechanism responsible for the association between RDW and adverse outcomes remains to be resolved, but could be related to disturbed iron metabolism or inflammation [19,25]. Because both FGF23 and RDW are independently associated with poor outcome measures, and both seem to be affected by iron, it is interesting to investigate whether a relation exists between FGF23 and RDW.

We hypothesized that a higher RDW is associated with more FGF23 cleavage, providing a common pathway in which both markers lead to adverse outcomes. Therefore, we examined the relationship between RDW and both intact and C-terminal FGF23 as well as the ratio between the two, and the difference between cFGF23 and iFGF23, in a cohort of patients with chronic kidney disease and chronic heart failure. Analyses were adjusted for markers of renal function, iron status and inflammation.

## Methods

### Subjects

For the current study we performed a post hoc cross-sectional analysis of baseline data from patients enrolled in the EPOCARES study (The Mechanisms of Erythropoetin Action in the CardioRenal Syndrome, ClinicalTrials.gov NCT 00356733). The study design of the EPOCARES study has been published previously [26]. The study is being carried out in compliance with the Helsinki Declaration, and the protocol has been approved at each participating center by its internal review board. In short, the EPOCARES study is an open-label, prospective, randomized trial in which patients with CHF, CKD (glomerular filtration rate 20–70 ml/min) and mild anemia (hemoglobin 10.3–12.6 g/dL in men, and 10.3–11,9 g/dL in women) were included to test the erythropoietic and non-erythropoietic responses to low-dose ESA treatment. Patients with active systemic disease as a cause of CHF or CKD were excluded. Other exclusion criteria were ESA therapy in the previous 6 months, bleeding, chronic inflammatory disease or malignancy. In all patients, standard treatment was started, comprising oral iron suppletion (ferrofumarate), calcium carbonate, aspirin when indicated and maximal tolerated dosages of a β-blocker, an angiotensin-converting enzyme (ACE) inhibitor or an angiotensin receptor blocker, according to CHF guidelines. Included patients were randomized into 3 groups: 1 group received a fixed dose of 50 IU/kg per week EPO to increase hemoglobin level to a maximum of 13.7 g/dL for men and 13.4 g/dL for women; another group was treated with 50 IU/kg per week EPO maintaining baseline hemoglobin levels for the first 6 months by phlebotomy. The control group received standard care without EPO. This translational study was designed primarily to discern hematopoietic from nonhematopoietic effects of erythropoietin (EPO) in cardiorenal patients. All baseline data were derived prior to randomization and initiation of EPO treatment.

The original study population of the EPOCARES study consisted of 62 patients. Five patients withdrew their informed consent and one patient was excluded because of malignancy diagnosed after inclusion. Baseline RDW data were missing for two patients and two outliers of FGF23 were excluded, since these values exceeded the third quartile by a magnitude greater than 1.5 (IQR).

### Biochemical Analysis

Biochemical measurements were performed at baseline and blood samples were drawn between 8 and 9 AM in supine position and stored immediately at -80°C until analysis.

Levels of Hb, hematocrit, MCV and RDW were measured using a Sysmex XE-2100 hematology analyzer (Toa Medical, Kobe, Japan). Plasma interleukin-6 (IL-6) levels were measured in duplo using a commercially available ELISA kit (R&D Systems, Minneapolis, USA).

As a marker of iron stores [27], ferritin was determined using a sandwich immunoassay on an Acces 2 immunoanalyzer within a Dx automated system from Beckman Coulter (Brea, CA). Function iron availability was determined by measuring transferrin saturation (TSAT) and was calculated from serum iron and transferrin estimates obtained with standard methods on a Beckman Coulter Dx. Renal function was estimated by means of MDRD. Reference values of all parameters are shown in Table 1.

**Table 1:** Main clinical and biochemical characteristics of patients from the EPOCARES study at baseline.

Characteristics*	All patients n = 52	Reference values
Age (yrs)	73 [69–80]	
Male sex, n (%)	33 (63.5%)	
Smoking (%)	11.5%	
BMI kg/m²	25.9 [23.7–29.9]	
Diabetes Mellitus	36.5%	
Hypertension	78.8%	
Hemoglobin (g/dL)	11.8 ± 0.9	12.5–16.1 g/dL (f)
		13.7–17.0 g/dL (m)
Hematocrit (L/L)	0.35 ± 0.03	0.36–0.48 L/L (f)
		0.40–0.52 L/L (m)
MCV (/μm³)	90 ± 4	80–102
RDW (%)	14.1 ± 1.2	10.4–13.0%
MDRD (ml/min/1.73m²)	35 ± 14	> 60 ml/min/1.73m²
NT-proBNP (pg/mL)	1387 [688–2370]	<738 pg/ml
Ferritin (ng/mL)	129 [75–179]	10–200 ug/l
Iron (μmol/L)	10 [8.8–14]	9–30 umol/l
TSAT (%)	20 [16.3–25]	< 45%
CRP (mg/L)	5 [2–11.3]	0–10 mg/l
IL-6 (pg/mL)	3.27 [1.9–5]	< 10 pg/mL
iFGF23	107.3 [65.1–162.2]	20–50 pg/ml
cFGF23	197.5 [110–408.5]	< 125 RU/ml
iFGF23/cFGF23	0.803 [0.37–0.86]	
PTH	10.4 [6.7–15]	1.5–7 pmol/l
Phosphate	1.15 [1–1.2]	0.80–1.45 mmol/l

BMI = body mass index, MCV = mean corpuscular volume, RDW = red cell distribution width, MDRD = estimated glomerular filtration rate by modified diet in renal disease formula, NT-proBNP = N-terminal pro-brain natriuretic peptide, TSAT = transferrin saturation, CRP = C-reactive protein, IL-6 = interleukin 6, iFGF23 = intact fibroblast growth factor 23, cFGF23 = C-terminal fibroblast growth factor 23, PTH = parathyreoid hormone.

*values in mean ± standard deviation or median [interquartile range]

FGF23 was analyzed with two validated assays [28]. The iFGF23 was determined in serum using a sandwich ELISA, (Kainos Laboratories, Tokio, Japan), the intra- and interassay CV’s are <10% and <14%, respectively. The cFGF23 was assessed in EDTA-plasma using a sandwich enzyme-linked immunosorbent assay (ELISA) (Immutopics, San Clemente, CA, USA). The intra- and interassay CV’s are <5% and <16%, respectively. The former assay detects only the full-length FGF23, while the latter assay additionally measures the c-terminal fragments of truncated FGF23. In order to estimate the amount of intact FGF23 in relation to the total amount of FGF23 (i.e. intact FGF23 + C-terminal FGF23 as measured by the C-terminal assay), we calculated the iFGF23/cFGF23 ratio. We also estimated the absolute amount of C-terminal fragments by calculating the difference between total FGF23 and iFGF23.

### Statistical Analysis

Continuous variables at baseline were summarized as the mean ± standard deviation (SD) if normally distributed or otherwise as medians with interquartile range (IQR). Skewed variables were transformed to natural logarithms after which normality was checked again. After checking model assumptions, univariate linear regression analyses were used to test the relationship between iFGF23, cFGF23 and the difference between cFGF23 and iFGF23 (cFGF23-iFGF23) with RDW. The ratio iFGF23/cFGF23 was divided in tertiles because of violation of linearity and we used two dummies to estimate the regression coefficient between ratio iFGF23/cFGF23 and RDW. FGF23 was used as independent variable and RDW as dependent variable. To test the relationship between FGF23 and TSAT, univariate regression analysis was performed with TSAT as independent and FGF23 as dependent variable. Subsequently, four multivariable linear regression models were used to adjust for confounding of the primary analysis (RDW and cFGF23). A 10% change of the regression coefficient was considered to indicate relevant confounding. Model 1 adjusted for potential confounders of the relationship between cFGF23 and RDW derived from the literature [29–31]: eGFR, PTH, phosphate, BMI and smoking. Model 2 adjusted for variables used in model 1 and in addition markers of iron metabolism (TSAT and ferritin). Model 3 adjusted for variables used in model 1 and in addition markers of inflammation (IL-6 and CRP). In the final model 4, a combination of previous models was used to adjust for all possible confounders. Age and sex were ruled out to be effect modifiers. For statistical analysis, the SPSS software package version 20 was used (SPSS, IBM, Chicago, IL, USA).

## Results

### Population Characteristics

Demographics, baseline laboratory data and clinical characteristics of the 52 patients enrolled into this study are reported in Table 1.

Median age was 73 years (IQR 69–80) and 63.5% were male (Table 1). Of the population included in this study 11.5% were smokers and on average BMI was elevated (median 25.9, IQR 23.7–29.9). The mean RDW value was 14.1% ± 1.2, with a reference range of 10.4–13.0% and the mean eGFR 35 ± 14 ml/min/1.73m^2^. Both iFGF23 (median 107.3 pg/ml, IQR 65.1–162.2) and cFGF23 levels (median 197.5 RU/ml, IQR 110–408.5) were increased. CRP levels were only slightly elevated, showing that the study involved chronic stable patients in a relatively low-inflammatory state. Ferritin levels and TSAT were low-normal.

### Relation between FGF23 and TSAT

Univariate linear regression was performed to estimate the relation between FGF23 and TSAT. A statistically significant association was found between TSAT and cFGF23 (β = -12.35, P = 0.03), but not between TSAT and iFGF23 (β = -1.86, P = 0.31).

### Relation between FGF23 and RDW

**Fig 1:**
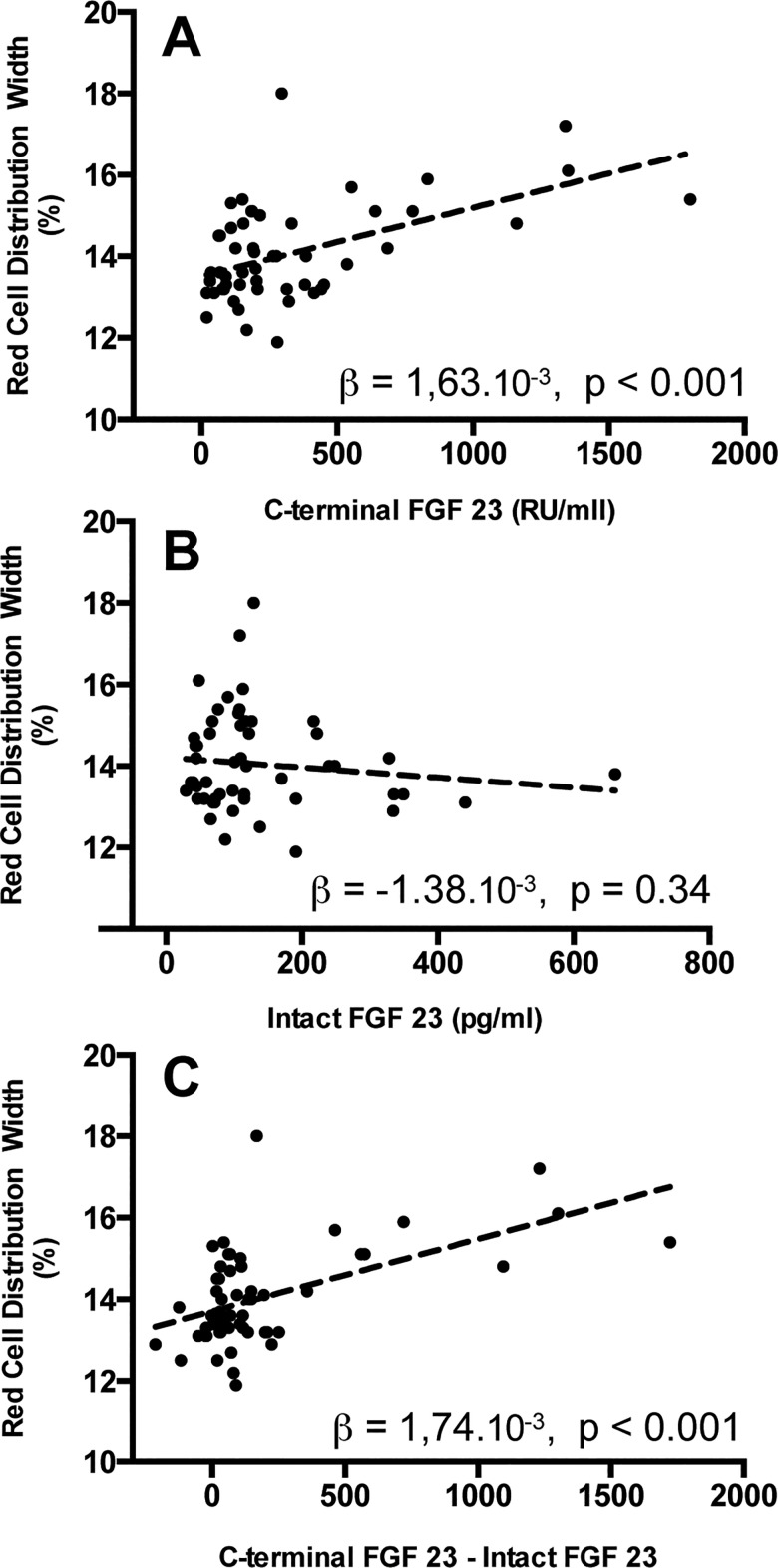
The relationship between baseline cFGF23 and RDW (A), baseline iFGF23 and RDW (B) and between cFGF23-iFGF23 and RDW (C).

Univariate linear regression showed a statistically significant relationship between cFGF23 and RDW (β = 1.63 x 10^−3^, P <0.001) in our population (Fig 1A), but not between iFGF23 and RDW (β = -1.38 x 10^−3^, P = 0.34, Fig 1B). The difference between cFGF23 and iFGF23 (cFGF23-iFGF23, representing the amount of c-terminal FGF23 fragments) was positively correlated with RDW (β = 1.74x10^-3^, P< 0.001, Fig 1C). In order to comply with the conditions for linear regression, we divided the iFGF23/cFGF23 ratio (representing the fraction of intact, biologically active FGF23) into tertiles. Both the second and the third tertile of the iFGF23/cFGF23 ratio were associated with RDW (β = -0.947, P = 0.014 and β = -1.253 and P = 0.002). This might be explained by reduced iron availability, as iron availability is a determinant of both RDW as well as the iFGF23/cFGF23 ratio due to increased cleavage of iFGF23 into C-terminal fragments in conditions of reduced iron availability.

To further analyze the relation of cFGF23 with RDW, we constructed several models to adjust for potential confounders (Table 2). Adjustment for eGFR, PTH, phosphate, BMI and smoking (model 1) did not modify the regression coefficient significantly between cFGF23 and RDW (β = 1.5 x 10^−3^, P = 0.001). Further adjustment for indicators of iron deficiency, ferritin and TSAT (model 2), marginally attenuated the association (β = 1.34 x 10^−3^, P = 0.003). Model 3, correcting for variables used in model 1 and for CRP and IL-6 as markers of inflammation, further attenuated the strength of the cFGF23-RDW association (β = 1.08x10^-3^, P = 0.023). After adjusting for all mentioned variables (model 4), the association between cFGF23 and RDW remained statistically significant (β = 0.969x10^-3^, P = 0.047).

**Table 2:** Multivariable linear regression for association between cFGF23 and RDW after adjustment for confounders.

Y = RDW	Regression coefficient	p-value
**Crude analysis**		
cFGF23 RU/ml	1.63 x 10^−3^	<0.001
Adjusted analysis 1*		
cFGF23 RU/ml	1.50 x 10^−3^	0.01
Adjusted analysis 2*		
cFGF23 RU/ml	1.34 x 10^−3^	0.003
Adjusted analysis 3*		
cFGF23 RU/ml	1.08 x 10^−3^	0.023
Adjusted analysis 4*		
cFGF23 RU/ml	0.97 x 10^−3^	0.047

1. Adjusted for eGFR, PTH, Phosphate, BMI and smoking

2. Adjusted for eGFR, PTH, phosphate, BMI, smoking, ferritin and TSAT

3. Adjusted for eGFR, PTH, phosphate, BMI, smoking, IL-6 and CRP

4. Adjusted for eGFR, PTH, phosphate, BMI, smoking, ferritin, TSAT, IL-6 and CRP

In a sensitivity analysis, further adjusted for hypertension, diabetes mellitus, hemoglobin level and 25-hydroxyvitamin D did not influence this relation (data not shown).

## Discussion

The main finding of this study is the strong and robust association between cFGF23 levels and RDW in patients with CKD and CHF, which persisted after adjustment of several potential confounders. Interestingly, in contrast with the consistent association between cFGF23 and RDW, no association between iFGF23 and RDW was observed. Our results are in line with previous observations connecting red cell properties with phosphate homeostasis [6,7]. As both red cell properties and calcium/phosphate homeostasis are important prognostic factors in CKD, detailed knowledge about their interaction could provide novel insights into the etiology of combined CHF and CKD and the subsequent deteriorated prognosis.

Currently, the underlying pathophysiological mechanisms linking FGF23 and RDW with outcome are unknown. Our data suggest an association between cFGF23 and RDW, raising the question whether there is an unknown factor that directly affects the risk of adverse outcome in combined CHF and CKD and whether this also affects both RDW and FGF23. Therefore, co-aggregation of changes in FGF23 and RDW caused by an established factor associated with renal function (i.e. potential confounding) should be ruled out. We adjusted for several potential confounders, known to influence FGF23 concentrations, based on the literature (eGFR, PTH, phosphate, smoking, and BMI) [29–31]: this did not substantially mitigate the strength of the association between RDW and FGF23.

Recent data demonstrated that iron deficiency can increase cFGF23, possibly as a result of increased FGF23 cleaving [6,7]. Wolf et al. demonstrated that iron deficiency stimulates FGF23 transcription whereby increased levels of iFGF23 are cleaved intracellularly into cFGF23 in healthy humans as such limiting its physiological effects on phosphate homeostasis. Therefore, iron metabolism may be a link between FGF23 and RDW in our patients with combined chronic heart and renal failure. Although we found a significant association between cFGF23 and TSAT, the association between cFGF23 and RDW was only marginally attenuated by TSAT and ferritin. This suggests that additional mechanisms could be involved. Of note, the study design of the EPOCARES study included oral iron supplementation in all groups.

In addition to iron metabolism, inflammation stands out as a potential mechanism explaining the association between FGF23 and RDW. Higher levels of FGF23 are independently associated with inflammation in patients with CKD [32] and high RDW values have been associated with plasma markers of inflammation in a large cohort of unselected adult outpatients [33] and in patients with heart failure [17]. Indeed, our subsequent analysis showed that the strength of the association between cFGF23 and RDW was attenuated, but not abolished, after adjusting for CRP and IL-6. Taken together, the association between cFGF23 and RDW may be partly explained by iron metabolism as well as by inflammation but remains statistically significant after adjustment for these factors, indicating that additional unknown factors may link cFGF23 and RDW.

Alternatively, cFGF23 may directly influence RDW. Although there is some debate, several studies demonstrate FGF23 effects on the vessel wall [34–36]. Endothelial responses to a high FGF23 level could induce suicidal red blood cell death (eryptosis) with a reactive rise in RDW [16,25]. As has been shown in animal model, the presence of c-terminal FGF23 fragments may modulate FGF23 mediated effects and as such the role of endothelial cells on erythrocyte turnover can be influenced [37].

Additional mechanistic studies linking iron metabolism, FGF23 and red cell fate are relevant in CKD given the high risks of cardiovascular disease and death in these patients. Future research in which FGF23 levels are manipulated in order to influence RDW could possibly lead to a potential therapeutic intervention and improve the cardiovascular outcome in CKD patients. Conversely, it may be of importance to investigate the effect of influencing RDW (via iron manipulation) on FGF23 production and cleavage in osteocytes. If indeed cFGF23 is toxic to vessels, targets to interfere in the FGF23 secretion and catabolism could be helpful in preventing cardiovascular diseases.

Limitations of the study as a result of sample size need to be acknowledged. The size of our cohort which was based on the EPOCARES study is relatively small; yet the observed association between cFGF23 and RDW was robust in multivariate analyses. Furthermore, this study, performed among elderly people with CKD and CHF, may not be generalized to the entire CKD population. Finally, no cause-effect relationship can be established from this study due to its cross-sectional nature. The assumption that c-terminal FGF23 has its effect on the vascular wall is purely speculative, so we consider this a hypothesis-generating study that serves to fuel future prospective studies.

Strong points of our study include the fact that we measured both C-terminal and intact FGF23, which allowed us to obtain specific information on FGF23 cleaving, and the fact that we adjusted our analyses for markers of iron status and inflammation. However, this also yields a limitation, as the comparison of the results of the two assays measuring cFGF23 and iFGF23 is difficult due to the use of different units. Since the proportion of FGF23 that is cleaved is unknown, cFGF23 can only be reported as unit/volume. The two assays may detect different FGF23 epitopes and therefore in biological systems the affinity for these respective epitopes may differ and explain limited linearity between these two ELISAs. This may apply in our EPOCARES subjects as well. However, our group has published results comparing the two assays in a range of concentrations and demonstrated a reasonable linearity of both assays [28]. We decided to use iFGF23:cFGF23 ratio as a measure for the amount of cleaved FGF23 present, in accordance with recommendations by others [15].

In conclusion, our study demonstrates an association between cFGF23 and RDW but not iFGF23, suggesting that RDW is linked with FGF23 cleaving. Although iron deficiency and inflammation are known determinants of RDW as well as FGF23 metabolism, these factors only partly explained the association between RDW and FGF23. Further research is warranted to address additional mechanisms driving the association between FGF23 and red cell metabolism, and particularly RDW, in patients with CKD and CHF.

## Supporting Information

S1 Dataset File containing the source data on which the results of this study are based. (XLSX)

## References

[1] Scrutinio D, Passantino A, Santoro D, Catanzaro R (2011) The cardiorenal anaemia syndrome in systolic heart failure: prevalence, clinical correlates, and long-term survival. Eur J Heart Fail 13: 61–67. hfq167 [pii]; 10.1093/eurjhf/hfq167 20858705

[2] Smith GL, Lichtman JH, Bracken MB, Shlipak MG, Phillips CO, DiCapua P, Krumholz HM (2006) Renal impairment and outcomes in heart failure: systematic review and meta-analysis. J Am Coll Cardiol 47: 1987–1996. S0735-1097(06)00488-8 [pii]; 10.1016/j.jacc.2005.11.084 16697315

[3] Shlipak MG, Fried LF, Cushman M, Manolio TA, Peterson D, Stehman-Breen C, Bleyer A, Newman A, Siscovick D, Psaty B (2005) Cardiovascular mortality risk in chronic kidney disease: comparison of traditional and novel risk factors. JAMA 293: 1737–1745. 293/14/1737 [pii]; 10.1001/jama.293.14.1737 15827312

[4] Lippi G, Targher G, Montagnana M, Salvagno GL, Zoppini G, Guidi GC (2008) Relationship between red blood cell distribution width and kidney function tests in a large cohort of unselected outpatients. Scand J Clin Lab Invest 68: 745–748. 794905563 [pii]; 10.1080/00365510802213550 18618369

[5] Zoccali C, Yilmaz MI, Mallamaci F (2013) FGF23: A Mature Renal and Cardiovascular Risk Factor? Blood Purif 36: 52–57. 000351001 [pii]; 10.1159/000351001 23735695

[6] Farrow EG, Yu X, Summers LJ, Davis SI, Fleet JC, Allen MR, Robling AG, Stayrook KR, Jideonwo V, Magers MJ, Garringer HJ, Vidal R, Chan RJ, Goodwin CB, Hui SL, Peacock M, White KE (2011) Iron deficiency drives an autosomal dominant hypophosphatemic rickets (ADHR) phenotype in fibroblast growth factor-23 (Fgf23) knock-in mice. Proc Natl Acad Sci U S A 108: E1146–E1155. 1110905108 [pii]; 10.1073/pnas.1110905108 22006328 PMC3219119

[7] Wolf M, Koch TA, Bregman DB (2013) Effects of iron deficiency anemia and its treatment on fibroblast growth factor 23 and phosphate homeostasis in women. J Bone Miner Res. 10.1002/jbmr.1923 23505057

[8] Bournier M, Tissot N, Mari S, Boucherez J, Lacombe E, Briat JF, Gaymard F (2013) Arabidopsis ferritin 1 (AtFer1) gene regulation by the phosphate starvation response 1 (AtPHR1) transcription factor reveals a direct molecular link between iron and phosphate homeostasis. J Biol Chem 288: 22670–22680. M113.482281 [pii]; 10.1074/jbc.M113.482281 23788639 PMC3829352

[9] Fliser D, Kollerits B, Neyer U, Ankerst DP, Lhotta K, Lingenhel A, Ritz E, Kronenberg F, Kuen E, Konig P, Kraatz G, Mann JF, Muller GA, Kohler H, Riegler P (2007) Fibroblast growth factor 23 (FGF23) predicts progression of chronic kidney disease: the Mild to Moderate Kidney Disease (MMKD) Study. J Am Soc Nephrol 18: 2600–2608. ASN.2006080936 [pii]; 10.1681/ASN.2006080936 17656479

[10] Hsu HJ, Wu MS (2009) Fibroblast growth factor 23: a possible cause of left ventricular hypertrophy in hemodialysis patients. Am J Med Sci 337: 116–122. 10.1097/MAJ.0b013e3181815498 ;00000441-200902000-00009 [pii]. 19214027

[11] Gutierrez OM, Januzzi JL, Isakova T, Laliberte K, Smith K, Collerone G, Sarwar A, Hoffmann U, Coglianese E, Christenson R, Wang TJ, deFilippi C, Wolf M (2009) Fibroblast growth factor 23 and left ventricular hypertrophy in chronic kidney disease. Circulation 119: 2545–2552. CIRCULATIONAHA.108.844506 [pii]; 10.1161/CIRCULATIONAHA.108.844506 19414634 PMC2740903

[12] Gutierrez OM, Mannstadt M, Isakova T, Rauh-Hain JA, Tamez H, Shah A, Smith K, Lee H, Thadhani R, Juppner H, Wolf M (2008) Fibroblast growth factor 23 and mortality among patients undergoing hemodialysis. N Engl J Med 359: 584–592. 359/6/584 [pii]; 10.1056/NEJMoa0706130 18687639 PMC2890264

[13] Jean G, Terrat JC, Vanel T, Hurot JM, Lorriaux C, Mayor B, Chazot C (2009) High levels of serum fibroblast growth factor (FGF)-23 are associated with increased mortality in long haemodialysis patients. Nephrol Dial Transplant 24: 2792–2796. gfp191 [pii]; 10.1093/ndt/gfp191 19395730

[14] Baia LC, Humalda JK, Vervloet MG, Navis G, Bakker SJ, de Borst MH (2013) Fibroblast growth factor 23 and cardiovascular mortality after kidney transplantation. Clin J Am Soc Nephrol 8: 1968–1978. CJN.01880213 [pii]; 10.2215/CJN.01880213 23929933 PMC3817902

[15] Wolf M, White KE (2014) Coupling fibroblast growth factor 23 production and cleavage: iron deficiency, rickets, and kidney disease. Curr Opin Nephrol Hypertens 23: 411–419. 10.1097/01.mnh.0000447020.74593.6f 24867675 PMC4322859

[16] Felker GM, Allen LA, Pocock SJ, Shaw LK, McMurray JJ, Pfeffer MA, Swedberg K, Wang D, Yusuf S, Michelson EL, Granger CB (2007) Red cell distribution width as a novel prognostic marker in heart failure: data from the CHARM Program and the Duke Databank. J Am Coll Cardiol 50: 40–47. S0735-1097(07)01270-3 [pii]; 10.1016/j.jacc.2007.02.067 17601544

[17] Forhecz Z, Gombos T, Borgulya G, Pozsonyi Z, Prohaszka Z, Janoskuti L (2009) Red cell distribution width in heart failure: prediction of clinical events and relationship with markers of ineffective erythropoiesis, inflammation, renal function, and nutritional state. Am Heart J 158: 659–666. S0002-8703(09)00551-1 [pii]; 10.1016/j.ahj.2009.07.024 19781428

[18] Pascual-Figal DA, Bonaque JC, Redondo B, Caro C, Manzano-Fernandez S, Sanchez-Mas J, Garrido IP, Valdes M (2009) Red blood cell distribution width predicts long-term outcome regardless of anaemia status in acute heart failure patients. Eur J Heart Fail 11: 840–846. hfp109 [pii]; 10.1093/eurjhf/hfp109 19696056

[19] Allen LA, Felker GM, Mehra MR, Chiong JR, Dunlap SH, Ghali JK, Lenihan DJ, Oren RM, Wagoner LE, Schwartz TA, Adams KF Jr. (2010) Validation and potential mechanisms of red cell distribution width as a prognostic marker in heart failure. J Card Fail 16: 230–238. S1071-9164(09)01176-2 [pii]; 10.1016/j.cardfail.2009.11.003 20206898 PMC3894681

[20] Tonelli M, Sacks F, Arnold M, Moye L, Davis B, Pfeffer M (2008) Relation Between Red Blood Cell Distribution Width and Cardiovascular Event Rate in People With Coronary Disease. Circulation 117: 163–168. CIRCULATIONAHA.107.727545 [pii]; 10.1161/CIRCULATIONAHA.107.727545 18172029

[21] Oh HJ, Park JT, Kim JK, Yoo DE, Kim SJ, Han SH, Kang SW, Choi KH, Yoo TH (2012) Red blood cell distribution width is an independent predictor of mortality in acute kidney injury patients treated with continuous renal replacement therapy. Nephrol Dial Transplant 27: 589–594. gfr307 [pii]; 10.1093/ndt/gfr307 21712489

[22] Patel KV, Ferrucci L, Ershler WB, Longo DL, Guralnik JM (2009) Red blood cell distribution width and the risk of death in middle-aged and older adults. Arch Intern Med 169: 515–523. 169/5/515 [pii]; 10.1001/archinternmed.2009.11 19273783 PMC2765040

[23] Patel KV, Semba RD, Ferrucci L, Newman AB, Fried LP, Wallace RB, Bandinelli S, Phillips CS, Yu B, Connelly S, Shlipak MG, Chaves PH, Launer LJ, Ershler WB, Harris TB, Longo DL, Guralnik JM (2010) Red cell distribution width and mortality in older adults: a meta-analysis. J Gerontol A Biol Sci Med Sci 65: 258–265. glp163 [pii]; 10.1093/gerona/glp163 19880817 PMC2822283

[24] Perlstein TS, Weuve J, Pfeffer MA, Beckman JA (2009) Red blood cell distribution width and mortality risk in a community-based prospective cohort. Arch Intern Med 169: 588–594. 169/6/588 [pii]; 10.1001/archinternmed.2009.55 19307522 PMC3387573

[25] Emans ME, van der Putten K, van Rooijen KL, Kraaijenhagen RJ, Swinkels D, van Solinge WW, Cramer MJ, Doevendans PA, Braam B, Gaillard CA (2011) Determinants of red cell distribution width (RDW) in cardiorenal patients: RDW is not related to erythropoietin resistance. J Card Fail 17: 626–633. S1071-9164(11)00153-9 [pii]; 10.1016/j.cardfail.2011.04.009 21807323

[26] van der Putten K, Jie KE, Emans ME, Verhaar MC, Joles JA, Cramer MJ, Velthuis BK, Meiss L, Kraaijenhagen RJ, Doevendans PA, Braam B, Gaillard CA (2010) Erythropoietin treatment in patients with combined heart and renal failure: objectives and design of the EPOCARES study. J Nephrol 23: 363–368. 59A56384-BA5A-41AD-91C7-179279C019CD [pii]. 20383871

[27] Wish JB (2006) Assessing iron status: beyond serum ferritin and transferrin saturation. Clin J Am Soc Nephrol 1 Suppl 1: S4–S8. 1/Supplement_1/S4 [pii]; 10.2215/CJN.01490506 17699374

[28] Heijboer AC, Levitus M, Vervloet MG, Lips P, ter Wee PM, Dijstelbloem HM, Blankenstein MA (2009) Determination of fibroblast growth factor 23. Ann Clin Biochem 46: 338–340. acb.2009.009066 [pii]; 10.1258/acb.2009.009066 19564163

[29] Vervloet MG, van Zuilen AD, Heijboer AC, ter Wee PM, Bots ML, Blankestijn PJ, Wetzels JF (2012) Fibroblast growth factor 23 is associated with proteinuria and smoking in chronic kidney disease: an analysis of the MASTERPLAN cohort. BMC Nephrol 13: 20 1471-2369-13-20 [pii]; 10.1186/1471-2369-13-20 22530966 PMC3366907

[30] Wolf M (2012) Update on fibroblast growth factor 23 in chronic kidney disease. Kidney Int 82: 737–747. ki2012176 [pii]; 10.1038/ki.2012.176 22622492 PMC3434320

[31] Mirza MA, Alsio J, Hammarstedt A, Erben RG, Michaelsson K, Tivesten A, Marsell R, Orwoll E, Karlsson MK, Ljunggren O, Mellstrom D, Lind L, Ohlsson C, Larsson TE (2011) Circulating fibroblast growth factor-23 is associated with fat mass and dyslipidemia in two independent cohorts of elderly individuals. Arterioscler Thromb Vasc Biol 31: 219–227. ATVBAHA.110.214619 [pii]; 10.1161/ATVBAHA.110.214619 20966399

[32] Munoz MJ, Isakova T, Ricardo AC, Xie H, Navaneethan SD, Anderson AH, Bazzano LA, Xie D, Kretzler M, Nessel L, Hamm LL, Negrea L, Leonard MB, Raj D, Wolf M (2012) Fibroblast growth factor 23 and Inflammation in CKD. Clin J Am Soc Nephrol 7: 1155–1162. CJN.13281211 [pii]; 10.2215/CJN.13281211 22554719 PMC3386678

[33] Lippi G, Targher G, Montagnana M, Salvagno GL, Zoppini G, Guidi GC (2009) Relation between red blood cell distribution width and inflammatory biomarkers in a large cohort of unselected outpatients. Arch Pathol Lab Med 133: 628–632. 2008-0279-OAR1 [pii]; 10.1043/1543-2165-133.4.628 19391664

[34] Yilmaz MI, Sonmez A, Saglam M, Yaman H, Kilic S, Turker T, Unal HU, Gok M, Cetinkaya H, Eyileten T, Oguz Y, Caglar K, Vural A, Mallamaci F, Zoccali C (2013) Longitudinal analysis of vascular function and biomarkers of metabolic bone disorders before and after renal transplantation. Am J Nephrol 37: 126–134. 000346711 [pii]; 10.1159/000346711 23391995

[35] Yilmaz MI, Sonmez A, Saglam M, Yaman H, Kilic S, Demirkaya E, Eyileten T, Caglar K, Oguz Y, Vural A, Yenicesu M, Zoccali C (2010) FGF-23 and vascular dysfunction in patients with stage 3 and 4 chronic kidney disease. Kidney Int 78: 679–685. ki2010194 [pii]; 10.1038/ki.2010.194 20613714

[36] Mirza MA, Larsson A, Lind L, Larsson TE (2009) Circulating fibroblast growth factor-23 is associated with vascular dysfunction in the community. Atherosclerosis 205: 385–390. S0021-9150(09)00009-4 [pii]; 10.1016/j.atherosclerosis.2009.01.001 19181315

[37] Goetz R, Nakada Y, Hu MC, Kurosu H, Wang L, Nakatani T, Shi M, Eliseenkova AV, Razzaque MS, Moe OW, Kuro-o M, Mohammadi M (2010) Isolated C-terminal tail of FGF23 alleviates hypophosphatemia by inhibiting FGF23-FGFR-Klotho complex formation. Proc Natl Acad Sci U S A 107: 407–412. 0902006107 [pii]; 10.1073/pnas.0902006107 19966287 PMC2806769

